# Association of the Lymphocyte-to-C-Reactive Protein Ratio with CT-Suspected Strangulation in Hospitalised Patients with Small Bowel Obstruction: A Retrospective Cohort Study

**DOI:** 10.3390/diagnostics16162601

**Published:** 2026-08-17

**Authors:** Hideya Itagaki, Jun Suzuki, Hirohito Metoki, Tomoyuki Endo

**Affiliations:** 1 Division of Emergency and Disaster Medicine, Tohoku Medical and Pharmaceutical University Hospital, Sendai 983-8512, Miyagi, Japan; entomo@hosp.tohoku-mpu.ac.jp; 2 Division of Infectious Diseases, Tohoku Medical and Pharmaceutical University Hospital, Sendai 983-8512, Miyagi, Japan; 3 Department of Infection Prevention and Control, Tohoku Medical and Pharmaceutical University Hospital, Sendai 983-8512, Miyagi, Japan; 4 Division of Infectious Diseases and Infection Control, Faculty of Medicine, Tohoku Medical and Pharmaceutical University, Sendai 983-8536, Miyagi, Japan; 5 Division of Public Health, Hygiene and Epidemiology, Faculty of Medicine, Tohoku Medical and Pharmaceutical University, Sendai 983-8536, Miyagi, Japan

**Keywords:** lymphocyte-to-C-reactive protein ratio, strangulated small bowel obstruction, intestinal ischaemia, C-reactive protein, emergency diagnosis

## Abstract

**Background:** Strangulated small bowel obstruction (SBO) is a time-critical emergency, and CT is not always available and is imperfectly accurate, prompting interest in blood-based markers such as the lymphocyte-to-C-reactive protein ratio (LCR). Whether LCR aids early risk assessment is unclear, because any signal may reflect earlier presentation (before CRP rises) or CT-based misclassification. **Methods:** In a single-centre retrospective cohort of adults hospitalised for SBO, strangulation was defined on contrast-enhanced CT; LCR was calculable in 142 of 200 patients (59 of 80 strangulated, 83 of 120 non-strangulated), and LCR-based analyses were complete-case. **Results:** A higher LCR was associated with strangulation (adjusted odds ratio 1.31 per log-unit, 95% CI 1.03–1.66; for an internally derived Youden cut-off of LCR > 0.269, adjusted odds ratio 2.42, 95% CI 1.05–5.55), but this attenuated to non-significance after adjustment for presentation time (2.23, 95% CI 0.95–5.21). Discrimination was poor (AUC 0.63): at the cut-off, sensitivity was 67.8% (95% CI 55.1–78.3), specificity was 55.4% (44.7–65.6), the positive likelihood ratio was 1.52 (1.13–2.05), and the negative likelihood ratio was 0.58 (0.38–0.88). When markers were oriented consistently, LCR did not discriminate better than CRP alone. **Conclusions:** An elevated LCR was associated with CT-suspected strangulation but showed poor discriminative power; the proposed threshold is exploratory and requires independent validation rather than clinical use.

## 1. Introduction

Mechanical small bowel obstruction (SBO) is a common surgical emergency, most frequently caused by postoperative adhesions and, less commonly, by hernias, neoplasms, or inflammatory disease [1,2]. Its most serious complication is strangulation with intestinal ischaemia, which may progress to necrosis, perforation, sepsis, and death unless surgery is performed promptly [3]. Early identification of strangulation is therefore essential. Contrast-enhanced computed tomography (CT) is the standard imaging modality and may demonstrate findings suggestive of ischaemia, including reduced bowel-wall enhancement, mesenteric oedema, closed-loop configuration, and free fluid [4,5]. However, CT is not always immediately available, and its accuracy for intestinal ischaemia is imperfect and protocol-dependent, with individual non-vascular signs unreliable in isolation and variable across readers and studies [6].

These limitations have prompted interest in inexpensive and routinely available blood-based markers. Composite indices derived from the white-cell differential, C-reactive protein (CRP), and albumin—including the neutrophil-to-lymphocyte ratio (NLR), platelet-to-lymphocyte ratio (PLR), CRP-to-albumin ratio (CAR), and lymphocyte-to-CRP ratio (LCR)—have been investigated across a range of acute and chronic conditions [7,8,9,10,11,12,13,14,15]. LCR in particular has been studied as a prognostic or diagnostic marker across malignant, surgical, and acute inflammatory conditions [16,17,18]. However, its diagnostic value in acute SBO remains uncertain, particularly during the early phase of the disease.

The interpretation of LCR in acute SBO may be influenced by the timing of presentation because CRP concentrations vary with the interval between symptom onset and blood sampling. Indeed, CRP begins to rise only 4–6 h after inflammation onset [19], so an early sample may show low CRP—and hence a high LCR—that reflects the timing of sampling rather than a lower disease burden. In addition, CT-based classification may be subject to misclassification when surgical confirmation is unavailable. These factors should therefore be considered when evaluating the association between LCR and strangulation.

Accordingly, we examined whether LCR was associated with CT-suspected strangulated SBO, whether this association persisted after adjustment for symptom-to-presentation time, and how it behaved when the analysis was restricted to surgically confirmed cases. We also compared the discriminative performance of LCR with that of other routinely available blood-based indices, with all markers oriented in the same direction. Because any threshold was derived within the same cohort, the accuracy analysis is exploratory, and we did not test whether LCR adds value beyond routine clinical and imaging assessment. As all patients were classified by contrast-enhanced CT, these data cannot address settings in which CT is unavailable.

## 2. Materials and Methods

### 2.1. Study Design and Setting

This was a single-centre, retrospective, observational study conducted at Tohoku Medical and Pharmaceutical University Hospital. We reviewed consecutive adults who were admitted through the emergency department (ED) with small bowel obstruction (SBO) between January 2020 and September 2024. The Ethics Review Committee for Life Science and Medical Research of Tohoku Medical and Pharmaceutical University approved the study (Approval No. 2024-6-075-0000), and the requirement for informed consent was waived because of its retrospective design. The study was conducted in accordance with the Declaration of Helsinki, and reporting follows STARD 2015 guidance [20].

### 2.2. Participants

We included patients aged ≥ 18 years who presented to the ED and were hospitalised for small bowel obstruction, defined clinically and confirmed on abdominal contrast-enhanced CT. We excluded patients who were not admitted, those aged < 18 years, and pregnant patients, as well as patients with obstruction due to colorectal malignancy, inflammatory bowel disease, or nephrotic syndrome, and patients receiving chemotherapy. We also excluded patients with established perforation, concurrent infection (e.g., pneumonia), or bacteraemia to study a cohort in which CRP would reflect the obstructive process itself rather than an independent septic or inflammatory source. This exclusion was a deliberate design choice, whose implications for case mix and selection bias are considered in the Discussion.

### 2.3. Definition and Classification of Strangulation

Strangulated SBO was diagnosed on contrast-enhanced CT primarily from a closed-loop configuration—the anatomical hallmark of mechanical strangulation—present in most cases, together with signs of intestinal ischaemia or necrosis (reduced or absent bowel-wall enhancement, mesenteric oedema or haziness, localised ascites, and pneumatosis intestinalis or portal-venous gas). In a minority without a definite closed loop, the diagnosis rested on these ischaemic features. Patients with a simple obstruction and neither a closed loop nor ischaemic features were classified as non-strangulated. Contrast-enhanced CT was interpreted as part of routine clinical care by the attending specialist—an emergency physician, gastroenterologist, gastrointestinal surgeon, or radiologist—and the final classification was made by the gastrointestinal surgeon. Because this was a routine, non-central read rather than a blinded central re-evaluation, we refer to the outcome throughout as CT-suspected strangulation. Where an operation was performed, intraoperative findings were recorded and classified as non-viable (necrotic) bowel requiring resection or viable but ischaemic (reversible) bowel not requiring resection. Amongst the 80 patients with CT-suspected strangulation, 64 (80.0%) underwent surgery. Because CT features denote ischaemia and impending necrosis rather than strangulation per se, we performed a sensitivity analysis restricted to surgically confirmed cases; because the decision to operate depended on the same clinical and CT features, this is a selected analysis subject to partial verification bias rather than validation against a surgical gold standard. For external hernias, classification was based on objective ischaemic or operative findings rather than reducibility alone, and we also examined the effect of excluding external hernias.

### 2.4. Data Collection and Definitions

For each patient we recorded, from the blood sample obtained at presentation, within approximately one hour of emergency-department arrival (including in patients who subsequently underwent surgery): sex, age, body mass index (BMI), Charlson Comorbidity Index (CCI), presenting symptoms, vital signs, Glasgow Coma Scale, aetiology of obstruction, and laboratory values (white-cell count and differential, platelet count, D-dimer, lactate dehydrogenase, albumin, lactate, and CRP). Outcomes were bowel resection, in-hospital death, and length of stay. Symptom-to-presentation time was defined as the interval, in hours, from patient-reported symptom onset to ED arrival. Tachycardia was defined as a heart rate > 100 beats/min. CRP was measured by latex-enhanced immunoturbidimetry on a Roche automated clinical-chemistry analyser (Roche Diagnostics GmbH, Mannheim, Germany), with an institutional reference range of <0.14 mg/dL and a lower reporting limit of 0.00 mg/dL; values were recorded in mg/dL and expressed in mg/L for the ratio. The LCR was calculated as the absolute lymphocyte count (×10^3^/µL) divided by CRP (mg/L); no CRP value was zero, so the ratio was always defined, and no special handling of below-limit values was required. LCR could be computed in 142 of the 200 patients because the lymphocyte differential was unavailable in the remaining 58 (these patients had a total white-cell count but no differential); all LCR-based analyses were therefore complete-case. Although the LCR was neither calculated nor used clinically, the clinicians and surgeon who classified strangulation could see the underlying CRP and lymphocyte values; the reference standard was therefore not fully blinded to the index test.

### 2.5. Statistical Analysis

Analyses were performed with Stata/MP 18.0 (StataCorp, College Station, TX, USA); consistently oriented ROC/AUC analyses, DeLong comparisons of AUCs, bootstrap internal validation, and the multivariable models reported here were computed in Python 3.10.12 (NumPy 2.2.6). Distributions of continuous variables were examined with histograms and Q–Q plots; non-normally distributed variables were summarised as median (interquartile range [IQR]) and compared with the Mann–Whitney U test, and categorical variables with Pearson’s chi-squared test. LCR was analysed primarily as a continuous variable after log transformation, with non-linearity assessed using restricted cubic splines. Discriminative performance was assessed by the area under the receiver-operating-characteristic curve (AUC); all markers were oriented so that higher values indicated strangulation, and AUCs were compared using the DeLong method with confidence intervals for the differences. An LCR cut-off determined by the Youden index was treated as exploratory and internally validated by bootstrap resampling. Multivariable logistic regression, adjusted for age, sex, BMI, CCI, tachycardia, and pain, estimated the association between LCR and strangulation; we report the numbers of events and parameters, assessed collinearity (variance inflation factors) and calibration (Hosmer–Lemeshow test), and evaluated the incremental value of LCR beyond the clinical model using a likelihood-ratio test and a comparison of AUCs. Two secondary analyses were pre-specified: adding symptom-to-presentation time and re-estimation using the surgical reference standard. A subgroup analysis by presentation window (<8 h), with an interaction test, was exploratory and hypothesis-generating. LCR-based models used complete cases. A two-sided *p* < 0.05 was considered significant; no formal power calculation was performed.

## 3. Results

**Figure 1:**
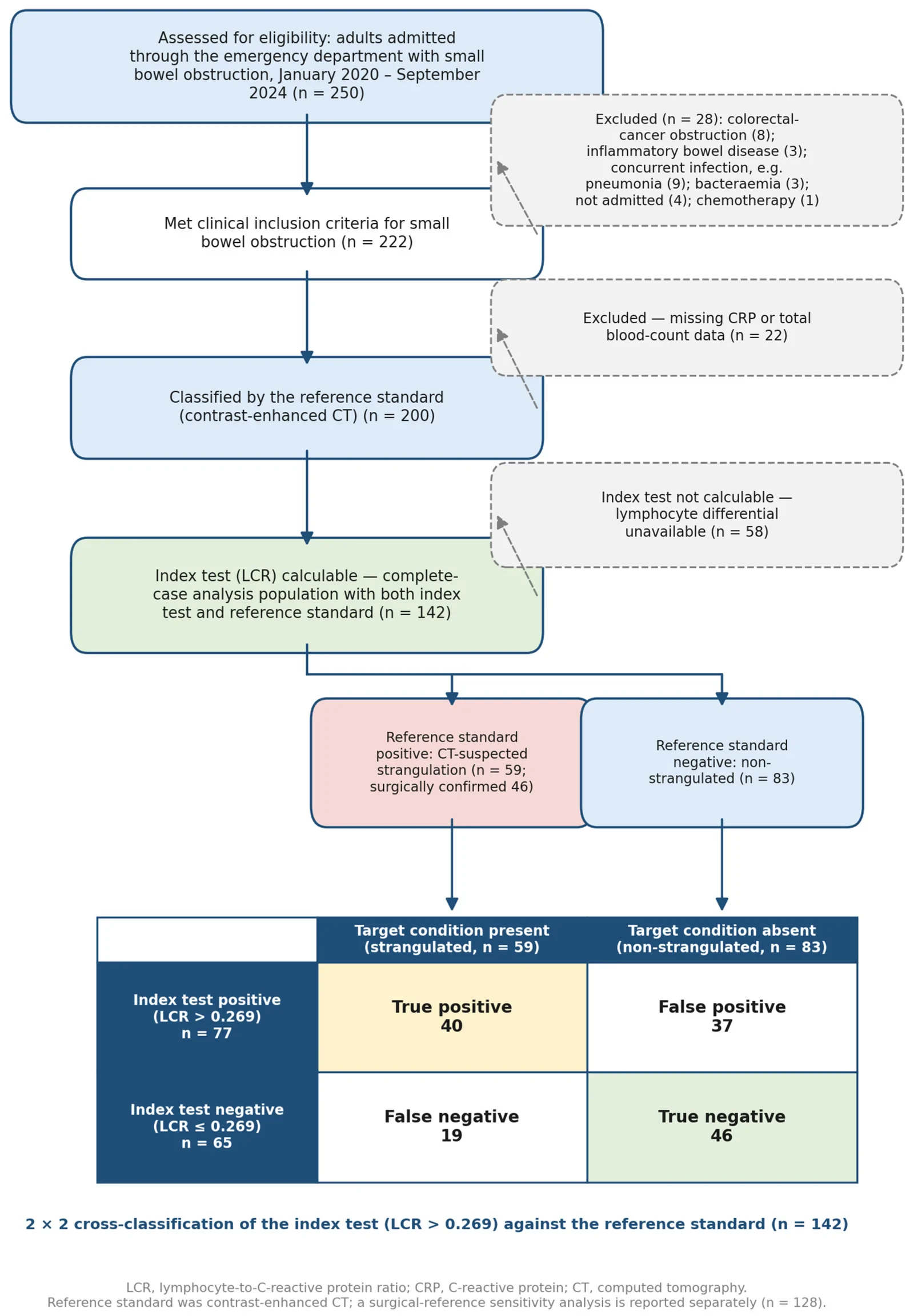
STARD 2015 [20] participant flow diagram. Of 250 patients assessed for eligibility, 28 were excluded (reasons shown), and a further 22 were excluded for missing CRP or total blood-count data, leaving 200 classified by the reference standard (contrast-enhanced CT). The index test (LCR) was calculable in 142 (strangulated 59, non-strangulated 83), who formed the complete-case analysis population; the lymphocyte differential was unavailable in 58. The lower panel cross-classifies the index test (LCR > 0.269) against the reference standard. CRP, C-reactive protein; CT, computed tomography; LCR, lymphocyte-to-C-reactive protein ratio. Created with Python 3.10.12 (Matplotlib 3.10.9).

During the study period, 250 patients presented to the ED with small bowel obstruction. After applying the exclusion criteria (*n* = 28) and excluding patients with missing CRP or total blood-count data (*n* = 22), 200 patients were included (Figure 1). Of these, 80 were classified as strangulated and 120 as non-strangulated on CT, and 64 of the 80 (80.0%) underwent surgery. Amongst the 200 included patients, a further 58 had a total white-cell count but no lymphocyte differential, so LCR was calculable in 142 (59 strangulated, 83 non-strangulated; prevalence 41.5%), and all LCR-based analyses were complete-case.

Patient characteristics are summarised in Table 1. The median age was 76 years (IQR 69–83). Female patients predominated in the strangulated group (66.3% vs. 43.3%, *p* = 0.001), and median BMI was higher in the strangulated group (21.6 vs. 19.7 kg/m^2^, *p* = 0.030); the Charlson Comorbidity Index did not differ. Strangulated patients tended to present earlier (median symptom-to-presentation time 7.5 vs. 10 h, *p* = 0.060). Abdominal pain was more frequent in the strangulated group (95.0% vs. 85.7%, *p* = 0.027). Heart rate differed between groups (*p* = 0.005), although the proportion with tachycardia did not (6.3% vs. 13.3%, *p* = 0.109).

Amongst biomarkers, CRP was significantly lower in the strangulated group (1.2 vs. 2.6 mg/L, *p* = 0.011), as were the CRP-to-albumin ratio (0.030 vs. 0.069, *p* = 0.013) and the CRP-to-platelet ratio (*p* = 0.007). Conversely, the LCR was significantly higher in the strangulated group (0.556 vs. 0.181, *p* = 0.009).

**Figure 2:**
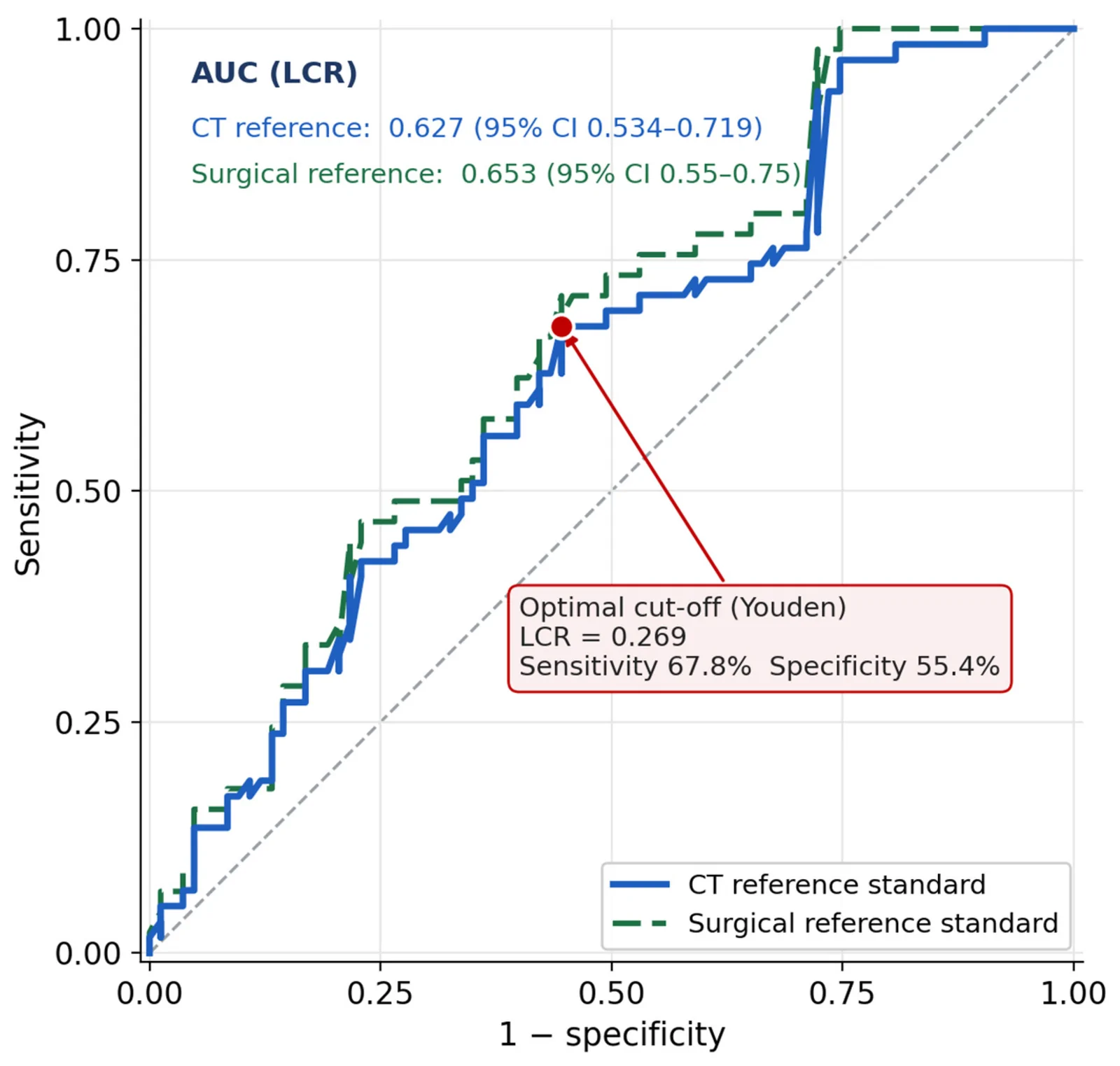
Receiver-operating-characteristic curves of the lymphocyte-to-C-reactive protein ratio (LCR) for strangulated small bowel obstruction, under a CT reference standard (AUC 0.627, 95% CI 0.534–0.719) and a surgical reference standard (AUC 0.653, 95% CI 0.55–0.75). The marked point is the Youden-optimal cut-off (LCR = 0.269; sensitivity 67.8%, specificity 55.4%). Created with Stata/MP 18.0.

Receiver-operating-characteristic analysis (Table 2 and Figure 2) showed that LCR discriminated strangulation only modestly (AUC 0.627, 95% CI 0.531–0.713). When all indices were oriented so that higher values indicated strangulation, discrimination was similar across CRP-based markers (CRP 0.627, CAR 0.626, and CRP-to-platelet ratio 0.639) and lower for the white-cell ratios (NLR 0.526, PLR 0.505); in pairwise DeLong comparisons, LCR did not differ significantly from any other index (all *p* > 0.4; Figure S1). The apparent superiority of LCR in an unoriented analysis arose because CRP, CAR, and the CRP-to-platelet ratio were lower in strangulated patients and therefore had AUCs below 0.5 as coded; once oriented, LCR conferred no advantage over CRP alone, and because lymphocyte counts did not differ between groups (*p* = 0.20), its signal is largely attributable to CRP. At the internally derived Youden cut-off of 0.269, LCR yielded a sensitivity of 67.8% (95% CI 55.1–78.3), specificity of 55.4% (44.7–65.6), PPV of 51.9% (40.9–62.8), NPV of 70.8% (59.0–80.3), a positive likelihood ratio of 1.52 (1.13–2.05), and a negative likelihood ratio of 0.58 (0.38–0.88) (Table 3; 2 × 2 counts 40 true-positive, 37 false-positive, 46 true-negative, 19 false-negative); on bootstrap resampling, this threshold was unstable (95% range 0.06–1.17). Added to a clinical model (age, sex, BMI, CCI, tachycardia, and pain; AUC 0.721), LCR produced only a small increase in discrimination (AUC 0.747; ΔAUC 0.026; likelihood-ratio *p* = 0.026 but DeLong *p* = 0.22).

In logistic-regression analysis (Table 4), an LCR > 0.269 was associated with strangulation in univariate analysis (odds ratio [OR] 2.70, 95% CI 1.33–5.48) and after multivariable adjustment (adjusted OR [aOR] 2.42, 95% CI 1.05–5.55). The association attenuated and was no longer significant after further adjustment for symptom-to-presentation time (aOR 2.23, 95% CI 0.95–5.21), and time itself was not an independent predictor of strangulation (OR 0.80, 95% CI 0.53–1.20). When the analysis was restricted to surgically confirmed strangulation, the association was preserved and stronger (aOR 3.52, 95% CI 1.37–9.04; AUC 0.66).

In secondary analyses, combining an LCR > 0.269 with pain was not significantly associated with strangulation after adjustment (aOR 2.22, 95% CI 0.95–5.18). In a pre-specified exploratory subgroup, the association appeared concentrated amongst patients presenting within 8 h of onset (aOR 7.01, 95% CI 1.47–33.5); however, the formal interaction test was not significant (*p* = 0.10), and the estimate was sensitive to the chosen threshold. Surgery was performed more often in the strangulated group (80.0% vs. 17.5%, *p* < 0.001), with no significant difference in in-hospital mortality or length of stay. Amongst the 85 operated patients, operative findings were non-viable (necrotic) bowel requiring resection in 17 (20.0%) and viable but ischaemic (reversible) bowel not requiring resection in 68 (80.0%); resection was performed in 16 of 64 with CT-suspected strangulation (25.0%, the remaining 48 having reversible ischaemia) and in 1 of 21 initially classified as non-strangulated on CT (4.8%). Aetiology differed markedly between groups (Table 1): bands, internal hernias, and volvulus occurred almost exclusively in strangulated patients, whereas 90% of non-strangulated patients had adhesions (*p* < 0.001). In a sensitivity analysis excluding the 25 external hernias (classified by irreducibility), the association was preserved (AUC 0.648; adjusted odds ratio for LCR > 0.269 2.77, 95% CI 1.12–6.86).

## 4. Discussion

In this emergency-department cohort, a higher LCR was associated with CT-suspected strangulation; the association attenuated and was no longer significant after adjustment for symptom-to-presentation time (adjusted OR 2.23, 95% CI 0.95–5.21), although it persisted in surgically confirmed cases. A reproducible association is not the same as accuracy, however: LCR discriminated strangulation poorly and, once markers were oriented consistently, no better than CRP alone, so we do not consider it ready for clinical use as a diagnostic or triage test.

The direction of our finding is opposite to that reported in oncology, where a low LCR marks worse outcomes [16], and it therefore requires careful explanation. Two related mechanisms are likely. First, CRP kinetics: strangulated patients presented earlier (median 7.5 vs. 10 h), and because CRP rises only 4–6 h after the onset of inflammation [19], blood was frequently sampled before CRP had fully risen, yielding lower CRP and hence higher LCR. Second, cohort composition: by design we excluded patients with perforation and bacteraemia—the most advanced, high-CRP presentations of strangulation—so the strangulated group that remained was enriched for earlier, less inflamed cases. Both mechanisms indicate that the absolute LCR values here are shaped by timing and case selection, and that LCR reflects an early, pre-CRP-surge window rather than a lower burden of disease. Framed as a design choice, this exclusion is deliberate rather than incidental: patients who have already progressed to perforation or bacteraemia proceed to surgery almost irrespective of any biomarker. Removing them therefore concentrates the analysis on the population in which risk assessment is genuinely uncertain.

These mechanisms raise the question of whether the LCR–strangulation association merely reflects early presentation. Adjustment for symptom-to-presentation time changed the point estimate only modestly (2.42 to 2.23), but the association was then no longer significant (95% CI 0.95–5.21, *p* = 0.065), and presentation time was not itself an independent predictor (OR 0.80); residual confounding cannot be excluded because symptom onset was patient-reported and the interval to blood sampling was not measured uniformly. Timing and case selection clearly influence the absolute CRP level, and we cannot firmly separate these from the association itself.

Concerns about the reference standard deserve equal attention. Strangulation was classified on contrast-enhanced CT and finally adjudicated by the gastrointestinal surgeon, a heterogeneous and non-central process susceptible to observer variability; we therefore describe the outcome as CT-suspected strangulation. To probe misclassification, we repeated the analysis restricted to the 64 surgically confirmed cases, in which the association was preserved and numerically stronger (aOR 3.52; AUC 0.66). This finding should be interpreted cautiously. Patients were operated on precisely because their clinical and CT findings suggested strangulation—the same features that define the outcome—so surgical confirmation is not independent of the diagnosis (partial verification bias). We therefore regard this as a selected sensitivity analysis, not validation against a surgical gold standard; it also cannot speak to strangulated patients managed without surgery.

Amongst the blood-based indices, LCR did not discriminate better than CRP, and an AUC of 0.63 with a positive likelihood ratio of only 1.52 corresponds to poor discrimination. We therefore make no claim that LCR can diagnose strangulation or guide the choice between conservative and operative management. Beyond a clinical model, LCR added little and non-significantly (ΔAUC 0.026); we did not evaluate whether it accelerates surgical consultation, improves real-world decisions, or adds value beyond CT. Because the lymphocyte differential was missing in 29% of this cohort, LCR was not universally available. Any potential role would be limited to generating, rather than confirming, suspicion of strangulation, and would require prospective evaluation. Although the Youden index identified 0.269 as the optimal cut-off in this cohort, the clinically appropriate threshold depends on the intended rule-in or rule-out role, disease prevalence, and the consequences of false-positive and false-negative classifications; the cut-off was also unstable on resampling and should be regarded as exploratory until independently validated [21].

In an exploratory, pre-specified subgroup analysis motivated by CRP kinetics, the association appeared concentrated amongst patients presenting within 8 h. We interpret this cautiously: the subgroup was small, the interaction test was not significant, and the finding was threshold-sensitive. It should be regarded as hypothesis-generating—consistent with the idea that CRP-based ratios are most informative before CRP rises—and requires prospective, timing-anchored confirmation. In a further exploratory analysis using the hardest endpoint, bowel resection (*n* = 17), LCR did not discriminate (median 0.34 vs. 0.36; AUC 0.52), whereas lactate, a direct marker of tissue ischaemia, was higher in resected patients. This dissociation is consistent with our central interpretation: LCR marks an early, pre-CRP-surge window of strangulation rather than established necrosis, for which direct ischaemia markers appear more informative. Given the small number of resections, this observation is hypothesis-generating only.

This study has several limitations. It is retrospective and single-centre, with attendant risks of selection bias and unmeasured confounding, and no formal sample-size calculation was performed. The reference standard was primarily CT, whose accuracy for strangulation is imperfect, and surgical confirmation was available in 80.0% of strangulated cases. Our exclusion of perforation and bacteraemia removed the most severe strangulated presentations and is itself a source of selection bias that likely contributed to the paradoxical CRP and LCR values. LCR could be calculated in 142 of 200 patients, so LCR-based analyses are complete-case; symptom-to-presentation time was patient-reported and approximate; blood was, however, sampled at presentation within about one hour of arrival in all patients (including those who proceeded to surgery), so the index test reflected a consistent, early time point, and the symptom-to-sampling interval closely tracked the symptom-to-presentation time we adjusted for. Prospective, multicentre studies using a surgical reference standard, anchored to time from symptom onset and retaining the full severity spectrum, are needed to validate whether LCR adds usable information in the early triage of strangulated SBO. Aetiology also differed strongly between groups (bands, internal hernias, and volvulus were largely confined to strangulated patients) and may have confounded the association, although the association persisted in a sensitivity analysis excluding external hernias.

## 5. Conclusions

In conclusion, a higher LCR was independently associated with CT-suspected strangulated small bowel obstruction, and our analysis clarifies the basis of this signal: it is largely driven by CRP and reflects an early, pre-CRP-surge window rather than a lower disease burden. These findings offer a timely, cautionary perspective for the expanding use of inflammatory ratios in acute care: with only modest discrimination, no advantage over CRP, and an internally derived, unstable threshold, LCR is not yet a clinical decision rule, and—because the cohort depended on contrast-enhanced CT—these data cannot speak to settings where CT is unavailable. They nonetheless provide a clear rationale and a design blueprint for prospective, multicentre studies that retain the full severity spectrum and anchor blood sampling to time from symptom onset.

## Figures and Tables

**Table 1:** Patient characteristics, biomarkers, and outcomes.

Parameter	All (*N* = 200)	Strangulated (*N* = 80)	Non-Strangulated (*N* = 120)	*p*
Clinical features				
Age, years, median (IQR)	76 (69–83)	75 (68–85)	76 (69–82)	0.829
Female sex, *n* (%)	105 (52.5)	53 (66.3)	52 (43.3)	0.001
BMI, kg/m^2^, median (IQR)	20.3 (17.6–22.8)	21.6 (18.2–23.7)	19.7 (17.6–21.7)	0.030
Charlson Comorbidity Index, median (IQR)	6 (4–7)	5 (4–7)	6 (4–8)	0.208
Symptom-to-presentation time, h, median (IQR)	9 (5–20)	7.5 (4–16)	10 (6–24)	0.060
**Aetiology, *n* (%)**				
Adhesions	135 (67.5)	27 (33.8)	108 (90.0)	<0.001
Band	18 (9.0)	18 (22.5)	0 (0)	
Internal hernia	14 (7.0)	14 (17.5)	0 (0)	
External hernia	25 (12.5)	17 (21.2)	8 (6.7)	
Volvulus	5 (2.5)	4 (5.0)	1 (0.8)	
Foreign body/bezoar	2 (1.0)	0 (0)	2 (1.7)	
Other/unspecified	1 (0.5)	0 (0)	1 (0.8)	
**Symptoms and vitals**				
Pain, *n* (%)	178 (89.0)	76 (95.0)	102 (85.7)	0.027
Nausea/vomiting, *n* (%)	110 (55.0)	41 (51.3)	69 (57.5)	0.384
Heart rate, bpm, median (IQR)	80 (68–91)	76 (65–89)	82 (72–92)	0.005
Tachycardia (>100 bpm), *n* (%)	21 (10.5)	5 (6.3)	16 (13.3)	0.109
**Biomarkers**				
Lymphocyte, ×10^3^/µL, median (IQR)	0.938 (0.702–1.321)	1.019 (0.705–1.400)	0.895 (0.698–1.264)	0.489
C-reactive protein, mg/L, median (IQR)	2.05 (0.50–6.65)	1.20 (0.40–4.95)	2.60 (0.70–7.00)	0.011
CRP-to-albumin ratio, median (IQR)	0.051 (0.012–0.178)	0.030 (0.009–0.138)	0.069 (0.016–0.213)	0.013
Neutrophil-to-lymphocyte ratio, median (IQR)	7.49 (4.40–10.53)	7.93 (4.40–9.96)	6.90 (4.31–11.02)	0.618
Lymphocyte-to-CRP ratio, median (IQR)	0.341 (0.081–1.365)	0.556 (0.106–2.075)	0.181 (0.058–0.827)	0.009
CRP-to-platelet ratio, median (IQR)	0.0094 (0.0026–0.0295)	0.0049 (0.0019–0.0189)	0.0130 (0.0034–0.0331)	0.008
**Outcomes**				
Surgery, *n* (%)	85 (42.5)	64 (80.0)	21 (17.5)	<0.001
In-hospital death, *n* (%)	1 (0.5)	0 (0)	1 (0.8)	0.417
Length of stay, days, median (IQR)	10 (7–14)	9 (7–13)	10 (7–15)	0.065

Biomarkers derived from the lymphocyte differential (lymphocyte count, neutrophil-to-lymphocyte ratio, and lymphocyte-to-CRP ratio) were available in 142 patients (strangulated 59, non-strangulated 83); the CRP-to-albumin ratio in 198 (strangulated 80, non-strangulated 118); the CRP-to-platelet ratio and all remaining variables in ≥197. Medians were computed on available cases. *p*-values are from the Mann–Whitney U test (continuous) or Pearson’s chi-squared test (categorical).

**Table 2:** Discriminative performance (ROC AUC) of blood-based indices for strangulated SBO.

Index	AUC	95% CI
Lymphocyte-to-CRP ratio (LCR)—CT reference	0.627	0.534–0.719
Lymphocyte-to-CRP ratio (LCR)—surgical reference	0.653	0.552–0.743
CRP-to-platelet ratio	0.611	0.532–0.691
C-reactive protein	0.605	0.526–0.685
CRP-to-albumin ratio (CAR)	0.604	0.523–0.684
Lactate dehydrogenase	0.577	0.496–0.657
Monocyte-to-lymphocyte ratio	0.576	0.478–0.673
White-cell count	0.568	0.487–0.649
Neutrophil count	0.568	0.472–0.664
Neutrophil-to-albumin ratio	0.550	0.453–0.648
Lymphocyte count	0.534	0.436–0.632
Neutrophil-to-lymphocyte ratio (NLR)	0.525	0.417–0.633
Platelet count	0.525	0.442–0.608
Lactate-to-albumin ratio	0.516	0.428–0.604
Lactate	0.514	0.427–0.602
Albumin	0.513	0.427–0.602
Platelet-to-lymphocyte ratio (PLR)	0.503	0.405–0.601
D-dimer	0.501	0.410–0.592

All indices are oriented so that higher values indicate strangulation; indices that were lower in strangulated patients (C-reactive protein, CRP-to-albumin ratio, CRP-to-platelet ratio, and D-dimer) were reversed, so their AUC equals one minus the as-measured value. AUCs were computed on complete cases (LCR, CT reference *n* = 142). The surgical-reference AUC (0.653, 95% CI 0.552–0.743) matches Figure 2. In pairwise DeLong comparisons, LCR did not differ significantly from any other index (all *p* > 0.4).

**Table 3:** Diagnostic performance of the LCR at the internally derived Youden threshold (LCR = 0.269) under the CT and surgical reference standards.

Measure	CT Reference (*n* = 142)	Surgical Reference (*n* = 129)
True positive, *n*	40	33
False positive, *n*	37	37
False negative, *n*	19	13
True negative, *n*	46	46
Prevalence	41.5%	35.7%
Sensitivity	67.8% (55.1–78.3)	71.7% (57.5–82.7)
Specificity	55.4% (44.7–65.6)	55.4% (44.7–65.6)
Positive predictive value	51.9% (40.9–62.8)	47.1% (35.9–58.7)
Negative predictive value	70.8% (59.0–80.3)	78.0% (65.9–86.6)
Positive likelihood ratio	1.52 (1.13–2.05)	1.61 (1.19–2.17)
Negative likelihood ratio	0.58 (0.38–0.88)	0.51 (0.31–0.84)
Area under the ROC curve	0.627 (0.531–0.713)	0.655 (0.554–0.743)

Surgical reference standard: strangulation defined by surgical confirmation. Of the 142 complete-case patients, 13 with CT-suspected strangulation who did not undergo surgery were excluded, leaving 129 (46 surgically confirmed strangulated, 83 non-strangulated); the adjusted surgical-reference model included 128 (one fewer, owing to a missing covariate); this is a selected sensitivity analysis subject to partial verification bias (see Methods). The threshold (0.269) was derived within this cohort and is exploratory.

**Table 4:** Association between an elevated LCR and strangulated SBO, with robustness to presentation timing and reference standard.

Analysis	OR/aOR (95% CI)	*p*	*n*
**Primary exposure—LCR > 0.269**			
Univariate	2.70 (1.33–5.48)	0.006	142
Multivariable—Model 1 *	2.42 (1.05–5.55)	0.038	140
Multivariable + presentation time—Model 2 †	2.23 (0.95–5.21)	0.065	140
presentation time (per log-h)	0.80 (0.53–1.20)	0.279	140
Surgical reference standard—Model 1 ‡	3.52 (1.37–9.04)	0.009	128
**Secondary—LCR > 0.269 combined with pain**			
Univariate	2.50 (1.24–5.03)	0.010	142
Multivariable §	2.22 (0.95–5.18)	0.064	140
**Exploratory subgroup—early presenters (<8 h)**			
LCR > 0.269, Model 1 (hypothesis-generating)	7.01 (1.47–33.5)	0.015	56
interaction LCR × early presentation	—	0.103	140

* Model 1: adjusted for age, sex, BMI, Charlson Comorbidity Index, tachycardia, and pain. † Model 2: Model 1 plus symptom-to-presentation time (per log-hour). ‡ Surgical reference standard: strangulation defined by surgical confirmation; a selected sensitivity analysis subject to partial verification bias (see Methods), Model 1 covariates. § Combined predictor: LCR > 0.269 together with the presence of pain; Model 1 covariates. OR, odds ratio; aOR, adjusted odds ratio; CI, confidence interval. The surgical-reference model includes 128 patients; the corresponding unadjusted 2 × 2 (Table 3) includes 129, the difference being one patient with missing BMI excluded from the adjusted model.

## Data Availability

The data presented in this study are available from the corresponding author on reasonable request.

## Supplementary Materials

The following supporting information can be downloaded at: https://www.mdpi.com/article/10.3390/diagnostics16162601/s1, Figure S1 (receiver-operating-characteristic curves for the lymphocyte-to-CRP ratio, C-reactive protein, the CRP-to-albumin ratio, and the CRP-to-platelet ratio, all oriented so that higher values indicate strangulation, showing near-identical discrimination; created with Python 3.10.12, Matplotlib 3.10.9) and the STARD 2015 checklist [20].

## Abbreviations

AUC, area under the curve; BMI, body mass index; CAR, C-reactive protein-to-albumin ratio; CCI, Charlson Comorbidity Index; CI, confidence interval; CRP, C-reactive protein; CT, computed tomography; ED, emergency department; IQR, interquartile range; LCR, lymphocyte-to-C-reactive protein ratio; LR, likelihood ratio; NLR, neutrophil-to-lymphocyte ratio; OR, odds ratio; PLR, platelet-to-lymphocyte ratio; ROC, receiver operating characteristic; SBO, small bowel obstruction.
