# Supplementary material for: Association of the Lymphocyte-to-C-Reactive Protein Ratio with CT-Suspected Strangulation in Hospitalised Patients with Small Bowel Obstruction: A Retrospective Cohort Study
Source: Diagnostics (Basel). 2026 Aug 17;16(16):2601. doi: 10.3390/diagnostics16162601 (PMC13511773; doi:10.3390/diagnostics16162601)
Supplement: Supplementary file 1 [file diagnostics-16-02601-s001.zip › Figure_S1.pdf]

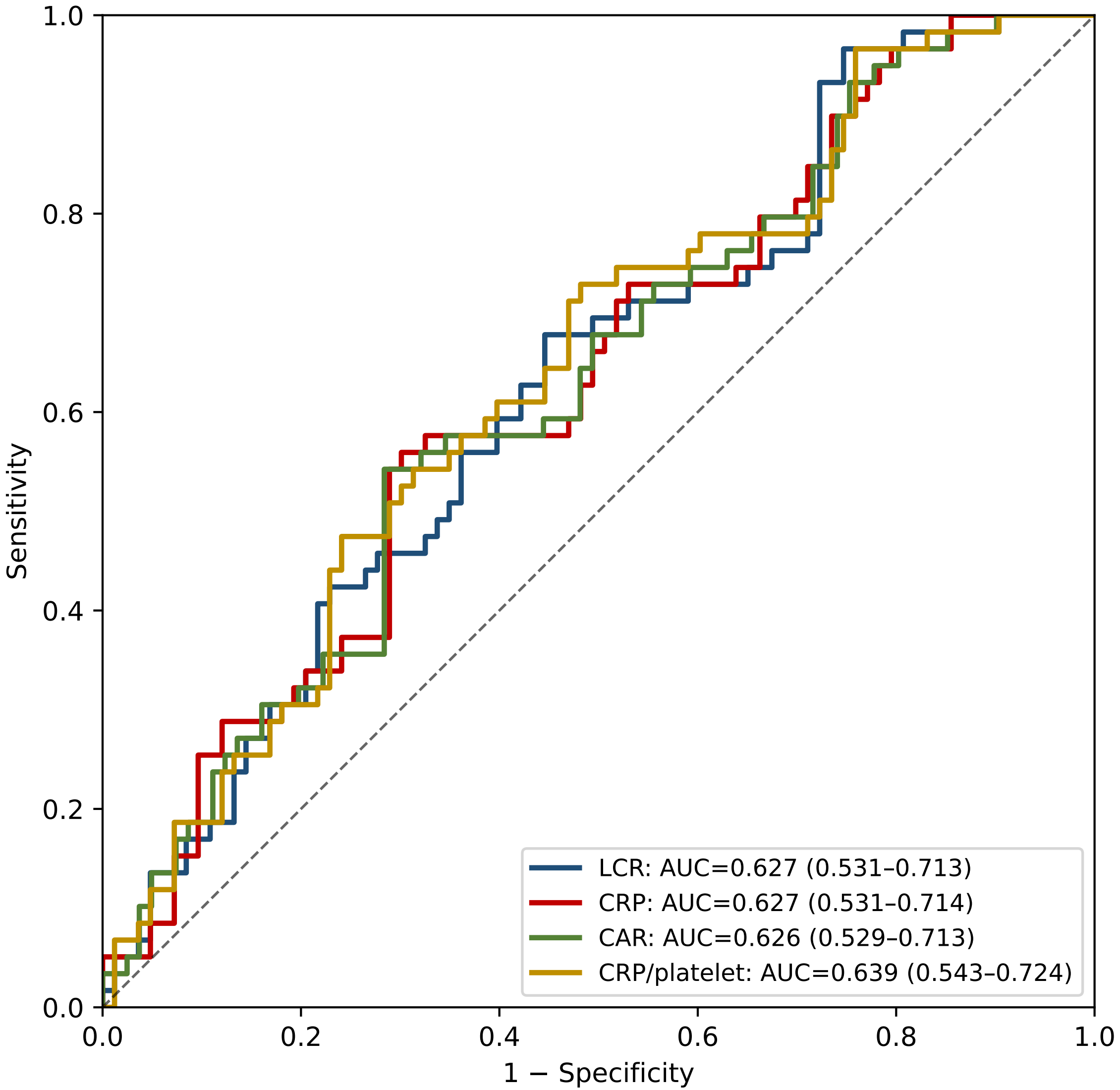

1.0
0.8
0.6
0.4
0.2
0.0
Sensitivity
0.0
0.2
0.4
0.6
0.8
1.0
1 − Specificity
LCR: AUC=0.627 (0.531–0.713)
CRP: AUC=0.627 (0.531–0.714)
CAR: AUC=0.626 (0.529–0.713)
CRP/platelet: AUC=0.639 (0.543–0.724)
