# Supplementary material for: Association of the Lymphocyte-to-C-Reactive Protein Ratio with CT-Suspected Strangulation in Hospitalised Patients with Small Bowel Obstruction: A Retrospective Cohort Study
Source: Diagnostics (Basel). 2026 Aug 17;16(16):2601. doi: 10.3390/diagnostics16162601 (PMC13511773; doi:10.3390/diagnostics16162601)
Supplement: Supplementary file 1 [file diagnostics-16-02601-s001.zip › STARD_2015_checklist.pdf]

# STARD 2015 Checklist

*Manuscript diagnostics-4497170 — Association of the Lymphocyte-to-C-Reactive Protein Ratio with CT-Suspected Strangulation in Hospitalised Patients with Small Bowel Obstruction: A Retrospective Cohort Study*

| No. | Item | Reported in section |
|---|---|---|
| **TITLE OR ABSTRACT** | | |
| **1** | Identification as a study of diagnostic accuracy using at least one measure of accuracy (such as sensitivity, specificity, predictive values, or AUC). | Title; Abstract |
| **ABSTRACT** | | |
| **2** | Structured summary of study design, methods, results, and conclusions. | Abstract |
| **INTRODUCTION** | | |
| **3** | Scientific and clinical background, including the intended use and clinical role of the index test. | Introduction (paragraphs 1–2) |
| **4** | Study objectives and hypotheses. | Introduction (final paragraph) |
| **METHODS** | | |
| **5** | Whether data collection was planned before the index test and reference standard were performed (prospective) or after (retrospective). | Methods 2.1 (retrospective) |
| **6** | Eligibility criteria. | Methods 2.2 |
| **7** | On what basis potentially eligible participants were identified (e.g., symptoms, previous test results, registry). | Methods 2.1–2.2 (ED admission for SBO, confirmed on CT) |
| **8** | Where and when potentially eligible participants were identified (setting, location, dates). | Methods 2.1 (single centre; Jan 2020–Sep 2024) |
| **9** | Whether participants formed a consecutive, random, or convenience series. | Methods 2.1 (consecutive adults) |
| **10a** | Index test, in sufficient detail to allow replication. | Methods 2.4 (LCR = lymphocytes $\times 10^3/\mu L$ ÷ CRP mg/L; assay details) |
| **10b** | Reference standard, in sufficient detail to allow replication. | Methods 2.3 |
| **11** | Rationale for choosing the reference standard (if alternatives exist). | Methods 2.3 (CT; surgical-reference sensitivity analysis) |
| **12a** | Definition of and rationale for test-positivity cut-offs of the index test, distinguishing pre-specified from exploratory. | Methods 2.5; Results (Youden 0.269, exploratory) |
| **12b** | Definition of and rationale for positivity categories of the reference standard, distinguishing pre-specified from exploratory. | Methods 2.3 (closed-loop–based CT-suspected strangulation) |
| **13a** | Whether clinical information and reference-standard results were available to the performers/readers of the index test. | Methods 2.4 (LCR an objective laboratory value; not read/interpreted) |
| **13b** | Whether clinical information and index-test results were available to the assessors of the reference standard. | Methods 2.4 (not formally blinded; CRP and lymphocyte values visible) |
| **14** | Methods for estimating or comparing measures of diagnostic accuracy. | Methods 2.5 (AUC, DeLong, logistic regression, bootstrap) |
| **15** | How indeterminate index-test or reference-standard results were handled. | Methods 2.4 (no CRP = 0; ratio always defined) |

| | | |
|---|---|---|
| **16** | How missing data on the index test and reference standard were handled. | Methods 2.4–2.5; Results; Figure 1 (complete-case; 58 missing differential) |
| **17** | Any analyses of variability in diagnostic accuracy, distinguishing pre-specified from exploratory. | Methods 2.5 (pre-specified secondary analyses; <8 h subgroup exploratory) |
| **18** | Intended sample size and how it was determined. | Methods 2.5 (no formal power calculation) |
| **RESULTS** | | |
| **19** | Flow of participants, using a diagram. | Figure 1 (STARD flow diagram) |
| **20** | Baseline demographic and clinical characteristics of participants. | Table 1; Results |
| **21a** | Distribution of severity of disease in those with the target condition. | Results (operative findings: necrosis vs reversible ischaemia); Table 1 (aetiology) |
| **21b** | Distribution of alternative diagnoses in those without the target condition. | Table 1 (aetiology: 90% adhesions in non-strangulated) |
| **22** | Time interval and any clinical interventions between index test and reference standard. | Methods 2.4; Discussion (sampling at presentation or immediately before surgery) |
| **23** | Cross-tabulation of the index-test results (or their distribution) by the reference-standard results. | Table 3; Figure 1 (lower panel, 2×2) |
| **24** | Estimates of diagnostic accuracy and their precision (e.g., 95% CI). | Table 3; Results (sensitivity, specificity, LRs, AUC with 95% CI) |
| **25** | Any adverse events from performing the index test or the reference standard. | Not applicable (retrospective; no test-related adverse events) |
| **DISCUSSION** | | |
| **26** | Study limitations, including sources of potential bias, statistical uncertainty, and generalisability. | Discussion (limitations paragraph) |
| **27** | Implications for practice, including the intended use and clinical role of the index test. | Discussion; Conclusions |
| **OTHER INFORMATION** | | |
| **28** | Registration number and name of registry. | Not applicable (retrospective observational study; not registered) |
| **29** | Where the full study protocol can be accessed. | No separate protocol; details available from the corresponding author |
| **30** | Sources of funding and other support; role of funders. | Funding statement (no external funding) |

*Adapted from: Bossuyt PM, Reitsma JB, Bruns DE, et al. STARD 2015: an updated list of essential items for reporting diagnostic accuracy studies. BMJ 2015;351:h5527. Items 28–29 marked in amber require author confirmation.*
